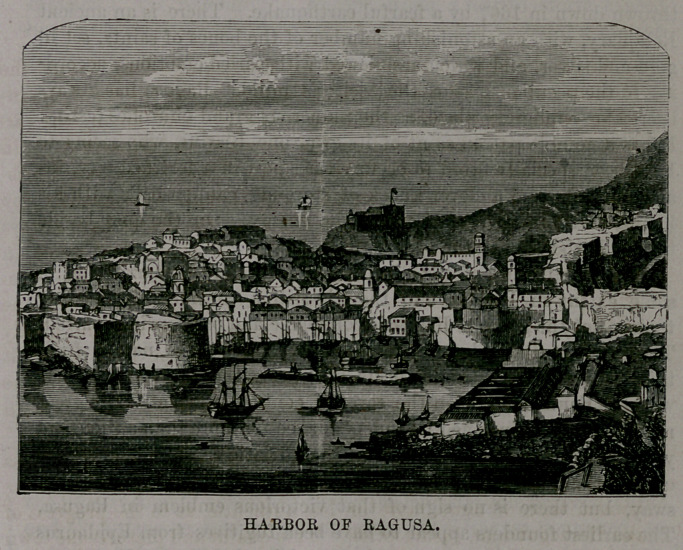# Ragusa

**Published:** 1876-08

**Authors:** Thos. Fredk. Ball


(*From Treasure Trove.*)

### *RAGUSA.*

---

Whilst the Eastern Empire was yet flourishing—long before the Moslem had planted his victorious banners on the towers of Byzantium—wild Slavic tribes wandered westward; and among the earliest were those who settled on the slopes of the Dinaric Alps, in the district now known as Herzegovina. These tribes were Christianized by the Eastern Church; and though subsequently the land became subject to Mohammedan sway, the Slaves have always upheld their Christianity. Herzegovina is about 220 miles in length by 50 in breadth, separated from the Adriatic by the Austrian province of Dalmatia, with two narrow outlets to the sea, one at Klex, and one at the Bocche di Cattaro. Austria would gladly have this little matter differently arranged, but the Turk makes it a religious principle never to cede voluntarily any territory that he has once acquired; and so for the present Dalmatia must remain broken into three fragments.

The central fragment of the three contains the town of Ragusa whose name has been so prominent of late as the head-quarters for the transmission of news, more or less authentic, as to the progress of the Herzegovinian insurrection. This quaint and picturesque town stands on a tongue of land at the foot of a barren mountain, from which a powerful modern fort commands the ancient city. The harbor of Ragusa, shown in our engraving, was, no doubt, admirably suited to the requirements of the vessels in which the city conducted its mediæval commerce; but it does not serve for the more capacious ships of modern times, and the real harbor is now at Gravosa, about a mile to the north-west. This is a safe and commodious port, surrounded with exquisitely beautiful scenery. Here formerly the wealthy Ragusans had their villas and gardens. A capital road, of French origin, since improved by the Austrians, conducts through a charming panorama of woods, and mountains, and orchards, and gardens, to the city.

The city of Ragusa, into which no horse or carriage is ever allowed to enter, is approached by drawbridges across dry moats, and through frowning gateways in the massive stone walls that encompass her round. These solid walls, of a cream-colored limestone, variegated with the tints of age, rise in many parts to the height of sixty or seventy feet. From north to south the town is traversed by the Corso, or Stradone, a really fine street, with a massive stone pavement. The houses, which are mostly in the Italian style, are of

HARBOR OF RAGUSA.

great strength and solidity; for Ragusa is subject to occasional earthquakes, and builds accordingly. All the other streets are narrow lanes, but quaint and artistic, with shops curiously hid away in arches, and everywhere exquisitely clean. About 8000 persons—a fifth of its ancient population—now reside in this intensely interesting city. Of the inhabitants, a recent writer says that "the men are splendid fellows in every physical respect; whilst the women, though amazingly strong and hardy, are plain, with rugged, roughhewn features, large muscular-development, and no figures to speak of."

Ragusa, with its graceful domes and campanili, its noble churches and public buildings, and its massive fortifications, presents a striking spectacle, either from the sea or the adjacent heights. The rocky environs are highly picturesque ; and those who do not object to an entire absence of level ground may make some delightful excursions in the vicinity, especially to the pretty Val d'Ombla, or Canosa, with its plane-trees. Inside the city, the interest of the public buildings is mostly architectural. For instance, there is the Dogana, Gothic without and Oriental within ; the Palace of the Rettore, in the Roman style, with Gothic details. The Cathedral is successor to the one built by Richard Cœur du Lion, which was thrown down in 1667 by a fearful earthquake. There is an ancient Reliquary, with an astonishing number of the bones of saints, set in gold and silver, and which are viewed with varied emotions, according to the peculiar bias of the visitor's mind. Ragusa has always been a very religious place, as the numerous grand churches testify. It has a notable patron saint of its own, one Blasius, who seems to have been specially fitted to occupy that post in a fortified city, inasmuch as he had a clever knack of stopping cannon-balls with his hands, and hurling them back at the enemy. On the quay beside the old harbor is a statue of this saint.

The manufacture of silk, leather, and rosaglio is carried on to some extent in Ragusa, and there is still a considerable coasting trade ; but the glory of Ragusa's commerce and prowess is departed. There was a time when its Ragosies, or Argosies (so named from this city), shared with the fleets of Venice the commerce of the world. Founded early in the seventh century, soon after its great rival, Ragusa maintained its independence for more than a thousand years as a free republic. All along the Adriatic coast, Venice carved on tower and wall the winged lion of St. Mark, as the symbol of her sway, but there is no sign of that victorious emblem in Ragusa. The earliest founders appear to have been fugitives from Epidaurus in Illyria, when that city was destroyed by the Slavi. Emigrants from various cities of Dalmatia and Albania soon joined the community. Fortifications were raised and a government established, which ultimately consisted of a general council of the leading families, a senate chosen by lot, and a Court or Rettore annually appointed. The inhabitants soon became renowned for their skill in shipbuilding and their maritime trade. They were divided into three orders—patricians, citizens, and plebeians. The former class were under little restraint, and their youth were particularly unruly. Razzi describes the grown up men as steady, well-behaved, just, and civil, but of the boys he says, "*Dalle mosche di Zara, e dai putti di Raugia cara, libera nos domine*" ("The Lord deliver us from the flies of Zara, and from the boys of our beloved Ragusa.")

For a few hundred years the history of Ragusa is a story of incessant wars and treaties with various powers. Croatians, Servians, Bosnians, Saracens, Bulgarians, emperors of Constantinople and Norman kings of Sicily, all had to be subdued or pacified according to circumstances, and through all the little state flourished and increased its trade, and its citizens grew rich and prospered.

One of the English kings became personally connected with Ragusa. After his fierce struggles with Saladin in the Holy Land, Richard I, was returning to Europe, when a fearful storm caused his lion-heart to sink within him, and he vowed to build a church on the spot where he should first land. He came on shore on the island of Lacroma, which, with its forts and villas, is now so conspicuous an object from the heights south of Ragusa. The Rettore of Ragusa, and some of the chief inhabitants hearing of his design, sent a deputation desiring him to build in their city instead of on the island, and promising to get a dispensation from the Pope for the alteration. Richard acceded to their request, borrowed a large sum of money according to his custom, which was increased by liberal donations from the inhabitants, and the cathedral was built. It was long noted for its regularity of design and beauty of ornament, till destroyed by the terrible earthquake of 1667. It was probably from this city that Richard set out disguised as Hugh the Merchant, on that ill-starred journey to Vienna, which resulted in his dreary captivity and romantic discovery by Blondel.

When in the fifteenth century the Turks established themselves in Europe, Ragusa found it desirable to purchase their forbearance by the payment of an annual tribute. During the sixteenth century the preservation of neutrality during the long struggle between Turks and Christians became an arduous task. Still Ragusa flourished, an outpost of civilization on the very borders of wild mountain tribes, and a city of refuge for fugitives and emigrants of every name. Ottomans or Christians flying from each other—Florentines mourning the fall of their proud city—Italians from all parts escaping from petty tyrants—all found at Ragusa a friendly welcome. In the seventeenth and eighteenth centuries the city found a special benefit from its friendship with the Ottoman power. Whilst ships of other states were almost swept from the Mediterranean by the pirates of Barbary, Ragusan barks passed untouched.

And so the grand old city remained an independent republic till

1806, when Napoleon I. played havoc with the free communities of Europe, and gave Ragusa as a Dukedom to Marshal Marmont. When Napoleon's conquests were divided amongst the allies it was incorporated with Austria.

Ragusa has long been noted for its commingling of Slavic vigor and Italian elegance. In the seventeenth century it was known as the Slavic Athens. It shows a long roll of citizens illustrious by their scientific or literary skill, from which we can only cull one or two names. Whilst Bacon and Shakespeare were thinking and writing in England, Marino Ghetadi, surnamed "The Demon of Mathematics," built himself a high European fame. In the eighteenth century the great Ragusan astronomer and mathematician Boscovich was entertained with all honor by the Royal Society at London. Cervano Tuberone and other writers have more than a local fame as historians. Amongst the native poets Gondola takes the first place. He was born in 1588 and died in 1638, his chief work being an Epic Poem, recounting the deeds of the Sultan Osman.

Our readers will see that Ragusa has a glorious past to look back upon. Its present is somewhat monotonous and dull, but still respectable. The future, who shall dare to forecast? From the Euxine to the Adriatic—forces, repressed for centuries, show signs of ferment, and if a young Slavonic empire is ever to spring into being, Ragusa may attain, and possibly exceed, its ancient influence and importance.

THOS. FREDK. BALL.

## Figures and Tables

**Figure f1:**